# Clinical and Molecular Traits of a Novel SPECC1L-ALK Fusion in a Patient with Advanced Non-Small Cell Lung Cancer

**DOI:** 10.3390/jpm14070670

**Published:** 2024-06-21

**Authors:** Antonella Centonza, Tommaso Mazza, Domenico Trombetta, Angelo Sparaneo, Francesco Petrizzelli, Stefano Castellana, Flavia Centra, Federico Pio Fabrizio, Concetta Martina Di Micco, Federica Benso, Fabrizio Tabbò, Luisella Righi, Alessandra Merlini, Paolo Graziano, Lucia Anna Muscarella

**Affiliations:** 1 Unit of Oncology, Fondazione IRCCS Casa Sollievo della Sofferenza, 71013 San Giovanni Rotondo, FG, Italy; a.centonza@operapadrepio.it (A.C.); c.dimicco@operapadrepio.it (C.M.D.M.); 2 Unit of Bioinformatics, Fondazione IRCCS Casa Sollievo della Sofferenza, 71013 San Giovanni Rotondo, FG, Italy; t.mazza@operapadrepio.it (T.M.); f.petrizzelli@operapadrepio.it (F.P.);; 3 Laboratory of Oncology, Fondazione IRCCS Casa Sollievo della Sofferenza, 71013 San Giovanni Rotondo, FG, Italy; d.trombetta@operapadrepio.it (D.T.); a.sparaneo@operapadrepio.it (A.S.); f.centra@operapadrepio.it (F.C.); fp.fabrizio@operapadrepio.it (F.P.F.); 4 Department of Oncology, San Luigi Gonzaga Hospital, University of Turin, 10043 Orbassano, TO, Italy; federica.benso@unito.it (F.B.); luisella.righi@unito.it (L.R.); alessandra.merlini@unito.it (A.M.); 5 SOC Oncologia ASLCN2 Alba e Bra, Ospedale Michele e Pietro Ferrero, 12060 Verduno, CN, Italy; 6 Unit of Pathology, Fondazione IRCCS Casa Sollievo della Sofferenza, 71013 San Giovanni Rotondo, FG, Italy; paologratz@gmail.com

**Keywords:** NSCLC, ALK, SPECC1L, gene fusion, target therapy, next-generation sequencing

## Abstract

Anaplastic lymphoma kinase (ALK) fusions account for 5–7% of non-small cell lung cancer (NSCLC) patients, the therapeutic approaches for which have significantly evolved in the last few years. However, the response to target therapies remains heterogeneous, partially due to the many different ALK fusion variants reported to date. Rare fusion variants have also been discovered, but their role in influencing responses to ALK inhibitors (ALKis) remains poorly elucidated. Laboratory investigation at both the tissue and protein levels, and a molecular profile by next-generation sequencing (NGS) were performed on a lung biopsy sample from one patient with poorly differentiated adenocarcinoma. An in silico prediction model using ColabFold software v1.5.5 was used to model and predict the entire structure of the chimeric aberrant ALK protein. Here, we report a case of a former smoker, a 60-year-old man, diagnosed with NSCLC and undergoing ALK translocation. He received first-, second- and third-generation ALK protein inhibitors (ALKis), showing a clinical benefit for about 4 years. IHC analysis and the molecular examination of the tissue biopsy indicated a positive staining for ALK and a novel *ALK* gene fusion variant, involving the sperm antigen with calponin homology and coiled-coil domain 1-like (SPECC1L) gene with an unreported breakpoint in exon 7. The novel SPECCL1::ALK fusion was identified using Anchored Multiplex PCR (AMP)-NGS technology and was predicted to retain the Pkinase_Tyr domain at the carboxy-terminal region of the resulting chimeric protein. To the best of our knowledge, this is the first case of an ALK-positive NSCLC patient carrying the SPECC1L exon 7 fusion breakpoint and one of the few reports about clinical outcomes related to SPECC1L::ALK fusion. The in silico hypothesized biological role of this newly identified fusion variant corroborates the observed clinical response to multiple ALKis. The molecular findings also reinforce the utility of AMP-based NGS technology as a valuable tool for the identification of rare chromosomal events that may be related to the variability of patient outcomes to different ALKis treatments.

## 1. Introduction

Around 5–7% of patients with advanced non-small cell lung cancer (NSCLC) carry an anaplastic lymphoma kinase (*ALK*) gene fusion, and younger, non-smoking female patients are primarily affected [1]. The aberrant activation of ALK requires a tyrosine kinase domain, and the translocation to partner genes can result in the ectopic and uncontrolled expression of the ALK protein, thus conferring sensitivity to ALK inhibitors (ALKis) [2].

Despite being a clinically validated therapeutic target, ALK fusions are not as accurately targeted as other carcinogenic drivers. Lung cancer has been linked to over 90 distinct ALK fusion partners [3]; however, compared to the most common ALK fusion variants, there is currently insufficient large-scale clinical evidence regarding the predictive value of unusual fusions [2,4]. ALK-TKI clinical use remains contingent upon *ALK* gene rearrangement detection, independently of the fusion partner’s molecular characteristics [5]. It is of note that clinical responses are heterogeneous, depending on the fusion subtype, according to recent scientific evidence in patients with ALK+ NSCLC [6], and this should impact the choice of ALKis in terms of adverse events and tolerability, which have gradually aroused attention [5]. Thus, for an informed therapeutic treatment, knowledge of how certain uncommon ALK fusions react to ALK-TKIs is required.

Sperm antigen with calponin homology and coiled-coil domain 1-like (*SPECC1L*) is a rare partner gene occasionally reported in ALK fusions of NSCLC, with only a few details known about its chimeric gene structure. By consequence, the sensitivity of rare SPECC1L::ALK fusion variants to ALKis remains uncertain and poorly investigated [7].

This is the first report on SPECC1L::ALK rearrangement in an NSCLC patient with a breakpoint in exon 7 of SPECC1L, which was identified using AMP-NGS technology and then investigated at the protein level by an in silico prediction model to clarify the pathogenicity of this rare event. Our results encourage the use of AMP-NGS and in silico prediction models to identify and clarify the potential oncogenic effects of rare ALK fusions in small patient cohorts that are less accessible for clinical follow-up monitoring.

## 2. Case Report

**Figure 1:**
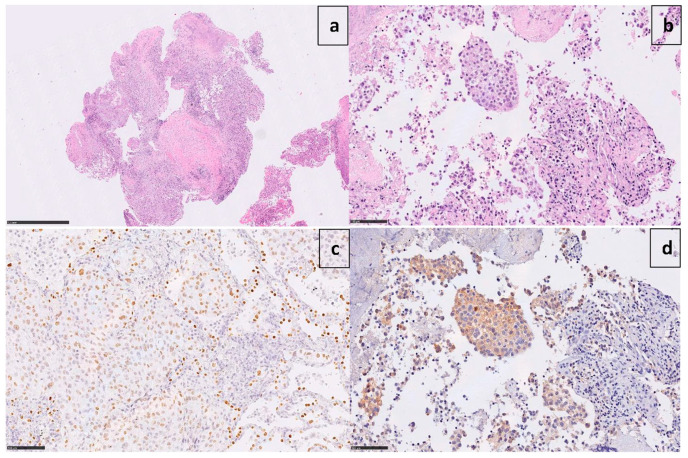
Microphotographs show poorly differentiated adenocarcinoma ((**a**) X1.7 H&E) with a predominant solid pattern ((**b**) X25 H&E), positive for TTF-1 ((**c**) X25) and strongly immunoreactive to ALK ((**d**) X25 D5F3 clone).

A 60-year-old Caucasian man (former smoker, 35 pack/year) was admitted in March 2017 to the Emergency Room of Fondazione IRCCS Casa Sollievo della Sofferenza in San Giovanni Rotondo (FG), Italy, for a persistent cough and chest pain. After hospitalization at the Internal Medicine Unit, a computed tomography scan (CT) revealed a 65 × 50mm mass between the superior and inferior lobes of the left lung, with pleural effusion. The involvement of the pre- and sub-carinal, bilateral axillary, and abdominal lymph nodes was also observed, as well as a left adrenal metastasis. A video-assisted thoracoscopic surgery (VATS) biopsy after a negative fibro-bronchoscopy showed a high-grade adenocarcinoma with a predominant solid pattern. Epidermal growth factor receptor (*EGFR*), Kirsten rat sarcoma viral oncogene homolog (*KRAS*), B-Raf proto-oncogene, and serine/threonine kinase (*BRAF*) genes were documented as wild-type. Immunoreactivity (3+ score) to ALK protein (clone D5F3, Cell Signaling Technology, Danvers, MA, USA) expression was documented (Figure 1).

**Figure 2:**
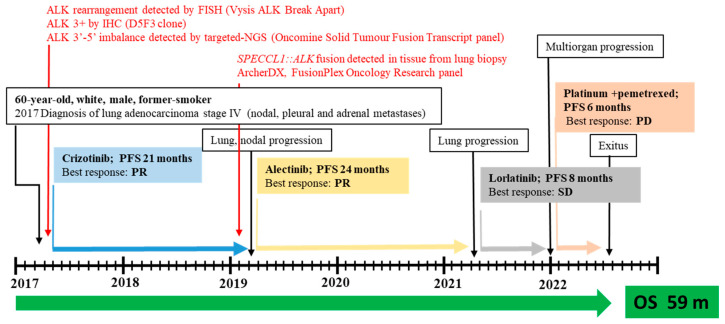
Timeline for patient with metastatic SPECCL1::ALK fusion who was treated with three subsequent lines of ALKis. Abbreviations: SD, stable disease; PR, partial response; PD, progression disease; PFS, progression-free survival; OS, overall survival; m, month; IHC, immunohistochemistry; FISH, fluorescence in situ hybridization; NGS, next-generation sequencing (for interpretation of the references to color in this figure legend, the reader is referred to the web version of this article).

*ALK* rearrangement was then confirmed in 28% (18/64) of neoplastic cells by fluorescence in situ hybridization (FISH) analysis using a Vysis ALK Break Apart FISH Probe Kit (IVD, Abbott Molecular), with an observed 5’ deletion in two nuclei. The patient was then eligible for targeted therapy with crizotinib as an upfront treatment, with disease stability for 21 months (Figure 2).

**Figure 3:**
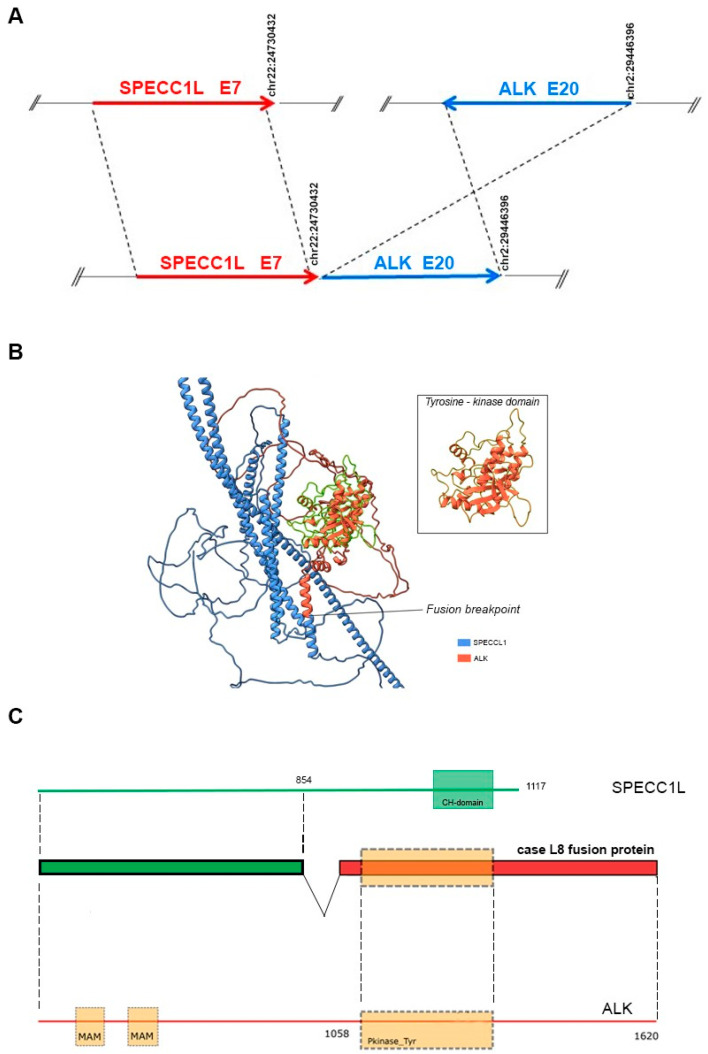
(**A**) Schematic representation of the novel ALK::SPECCL1 fusion gene variant identified (S7A20). The blue and red arrows at the bottom of the figure show the different gene blocks that form the chimeric transcript. The upper part of the figure shows, schematically, the breakpoints at the exonic level in the *ALK* and *SPECCL1* genes (GRCh37/hg19 reference sequence). Arrows sizes are not proportional to the exons involved in the rearrangements. (**B**) ColabFold model of the SPECCL1-ALK fusion protein. The SPECCL1 protein portion is stained in blue and the conserved ALK portion is stained in orange. The conserved ALK tyrosine-kinase domain is highlighted in green, and magnified into the box. (**C**) Schematic representation of the predicted SPECC1L-ALK fusion protein identified in the patient. The green portion represents part of SPECC1L protein conserved in the fusion protein, whereas the conserved portion of ALK protein is shown in red. The dashed rectangle represents the tyrosine kinase domain of ALK. The domains were mapped with the InterPro v88.0 program.

Lung and nodal progression were demonstrated during restaging by computed tomography (CT) in February 2019, so the patient was included in an ongoing clinical trial on the molecular profiling of NSCLC, approved by our local ethics committee (prot.171/CE). A large molecular screening was performed on the available diagnostic neoplastic formalin-fixed and paraffin-embedded (FFPE) specimen to refine his molecular profile. A first round of investigation into gene fusions was performed by RNA-targeted NGS analysis using the Oncomine Solid Tumour Fusion Transcript kit (Thermo Fisher Scientific, Waltham, MA, USA) with the Ion S5™ Sequencer System, and data were analyzed on the Ion Reporter Server (Thermo Fisher Scientific, USA), yielding strong evidence of an ALK fusion as a 5′-3′ probe imbalance, but without any fusion variant identification. To investigate the presence of a novel and previously untargeted fusion of *ALK*, the biological sample was therefore resequenced using the FusionPlex Oncology Research RUO panel (Archer DX Inc., Boulder, CO, USA) on the NextSeq500 platform (Illumina, San Diego, CA, USA), and the results were annotated through Archer™ Suite Analysis ver. 6.2.3. Identified fusions were classified as strong with, among others, the following settings: minimal reads for valid structural variation > 5; structural variation percent of GSP2 reads > 10. A rare and novel SPECCL1::ALK fusion with the recently identified *SPECCL1* partner gene [S7A20, chr22:24730541, chr2:29446394] was identified in the biological sample of the patient [7]. The novel identified SPECCL1::ALK fusion variant (S7A20) arises from fusion between exon 7 of SPECCL1 at an unreported breakpoint level and exon 20 of the *ALK* gene [S7 (SPECCL1)::A20 (ALK); chr22:24730541- chr2:29446394, GRCh37/hg19 release)] (Figure 3A).

**Figure 4:**
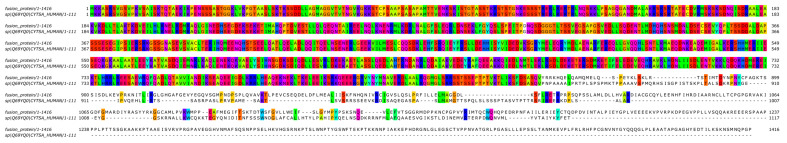
Bioinformatic alignment of the fusion protein with the wild-type SPECC1L protein (Uniprot Id: Q69YQ0). Colors indicate the presence of a match between the two sequences.

**Figure 5:**
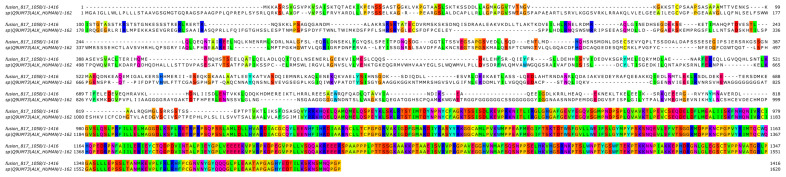
Bioinformatic alignment of the fusion protein with the wild-type ALK protein (Uniprot Id: Q9UM73). Colors indicate the presence of a match between the two sequences.

A two-stage bioinformatic analysis was performed to characterize this new variant, with the aim of obtaining a prediction of the chimeric protein structure, verifying the level of conservation of ALK’s functional domains, and therefore conserving the related biological functionality. We found that this novel SPECCL1::ALK (S7A20) fusion protein consisted of 1416 amino acids (aa) and, according to InterProScan [8], retained the intact tyrosine kinase domain of the ALK protein, which could result in constitutive kinase activity and oncogenic transformation, as reported for canonical ALK rearrangements (Figure 3B). This was confirmed by the alignment performed through the Clustal Omega tool (https://www.ebi.ac.uk/Tools/msa/clustalo/, accessed on 10th February 2024) of the sequence of the fusion protein onto the wild-type sequences of SPECCL1 (Figure 4) and ALK (Figure 5). This analysis identified uneven regions in terms of sequence similarity but, however, found the tyrosine kinase domain of the ALK protein completely preserved. This observation was also supported by the protein expression result from immunohistochemistry (IHC) analysis with a staining score of 3+. Data were processed using the Jalview v.2.11.20 software package.

We then predicted a model of the entire fusion product by resorting to the local implementation of the ColabFold software package [9], which was run on our local GPU-equipped high-performance computing (HPC) cluster [10,11] with standard settings. Paired and unpaired MSAs (but no template information) were given as the input. Finally, only the top-ranked structure was relaxed, using the standard minimization protocol already described in [12]. The model showed some unresolved regions, a long coiled-coil domain belonging to SPECCL1, and an intact ALK’s tyrosine kinase domain. The fusion breakpoint was identified in the middle of a newly structured alpha helix (Figure 3C).

After disease progression with crizotinib, the patient started a treatment with alectinib as a second-line treatment, which allowed control of the disease for an additional 24 months. A whole-body CT scan performed in March 2021 revealed an increased size of the pulmonary mass, prompting the start of lorlatinib therapy within a compassionate program. After 8 months, due to further multiorgan progression, platinum and pemetrexed chemotherapy was administered from December 2021 to May 2022. After the declining performance status of the patient, active therapy was discontinued and, in June 22, he died.

## 3. Discussion

Gene fusions are the result of complex genomic alterations that occur in tumor cells and often play an important role in the etiology of tumor progression and proliferation [13]. In clinical practice, gene fusions actually represent one of the most studied molecular markers, mainly in lung cancer patients, but also in other solid tumors [14,15,16]. In particular, the identification of *ALK*, *ROS1*, *RET*, and *NTRK1/2/3* gene rearrangements by the scientific community has given an enormous change in therapeutic perspective for patients suffering from NSCLC in the advanced stage of the disease [17]. These rearrangements, although presenting a low incidence, are in fact the basis of therapeutic strategies with molecularly targeted drugs [18]; therefore, they require particular attention aimed at clarifying their heterogeneity and establishing their effective oncogenic activity [19,20,21].

Many ALK-TKIs have been validated since the first EML4-ALK fusion in NSCLC was discovered, and their use in clinical practice has significantly improved PFS and OS in patients with metastatic ALK-positive NSCLC [22]. Nonetheless, there is heterogeneity in the clinical response between various ALK fusion subtypes, as well as ALK fusion variants [23]. A primary rationale for these observations is that distinct 5′ fusion partners may have a different impact on the biological features of ALK fusion proteins, resulting in variations in protein stability, expression levels, oncogenic potential, and ultimately, the response to ALK-TKIs [24]. Therefore, to increase the number of targetable ALK fusions and better understand their biological function, clinicians worldwide are urged to report all identified novel fusions and share information on fusion breakpoints and responses to ALK-TKIs [2].

According to diagnostic practices, the determination of *ALK* gene rearrangements has been performed for many years only using FISH and/or IHC techniques, which are low-cost methodologies that provide information on the integrity of the gene sequence or on the expression of the aberrant protein, respectively. But in contrast, these approaches do not give any information about the presence of chimeric transcripts or the related chimeric protein structure.

In this scenario, NGS represents a better complementary detection method due to its ability to view the breaking point and determine whether the fusion gene created has a normal biological function [25,26]. Precise recommendations exist today for applying NGS high-throughput platforms in routine clinical contexts [27]. However, its usage, interpretation, and harmonization with other diagnostic techniques are not always simple or largely accessible due to the quality of the RNA obtained from FFPE sections, which can generate false-negative results [28]. Moreover, the currently used NGS panels for fusion discovery only allow the detection of well-known fusion transcript variants and the 3′-5′ expression imbalance, but do not give any indication about existing unreported fusions. The recent implementation of NGS using AMP technology, accompanied by adequate data analysis, can certainly constitute a great advantage in overcoming some of these limits. Markedly, it could help to reduce the heterogeneity of results obtained from different technical approaches in order to identify those ALK+ patients with a true clinical benefit from treatment with specific targeted drugs, as well as to decodify ambiguous results obtained from discordant IHC/FISH and targeted RNA-NGS analyses. Moreover, the use of AMP technology should help the identification of complex and novel patterns of coexistent instances of ALK common with rare fusion variants, which could affect different ALKi responses [29]. As described in our work, the identification of the previously unreported SPECCL1::ALK gene fusion with a novel genetic breakpoint highlights how the approach based on AMP technology is preferable to the Ampliseq targeted RNA chemistry used to identify novel or rare gene fusions, particularly for those cases with a strong positive IHC [30].

Even though many rare ALK fusion variations have been discovered, it is challenging to analyze their differences in response to ALK-TKIs by directly assessing the clinical history of the few individuals who share the same unusual fusion variant.

The SPECC1L::ALK fusion was first mentioned in a Chinese patient with lung adenocarcinoma in 2019 [31], but no details about the clinical outcome of the patient were reported. To date, responses to ALKi in two NSCLC patients with different SPECC1L-AL fusion variants have been published only in one previous work, and the clinical outcome of the patients supports the idea that SPECC1L may be a tumor driver factor that can be inhibited by a series of TKIs [7].

In this context, the in silico analysis of the molecular structure and biochemical properties of fusion proteins could help to close this gap by evaluating the preservation of the spatial conformation of functional ALK protein domains [32,33,34]. ALK fusion variants lacking the ALK kinase domain are predicted to be resistant to certain ALKi therapies, according to a number of studies [35]. Furthermore, in order to enable the automated activation of kinases, fusion partners must supply dimerization domains in order to automatically activate and send a signal downstream, just like other tyrosine kinases [36]. It is finally reported that some specific variants of ALK fusions are null fusions, meaning they are unable to translate tumor-causing kinesins [37].

We did not establish an in vitro cell model for this specific ALK fusion variant to confirm the oncogenicity of this novel variant or test the response of the different ALKis used. Anyway, the results of our bioinformatic analysis predicting the structure of the putative SPECCL1-ALK chimeric protein revealed that the tyrosine kinase domain of ALK remains conserved, thus indicating a most likely oncogenic role for the aberrant protein. This hypothesis is also corroborated by an IHC score of 3+ and a favorable outcome of the patient who received crizotinib, alectinib, and lorlatinib as sequential ALKi treatments, achieving disease control lasting 54 months. The 3D model obtained from our computational analysis supports the idea that investigating the oncogenicity of aberrant fusion proteins by in silico protein domain analysis should be of great support to predict the efficacy of different inhibitors to block the activity of different gene fusions, thus giving an indirect indication about response to specific treatments. This is of particular interest in the case of rare fusion variants whose responses to ALKi treatment could not be monitored in real life in a large cohort of patients.

## 4. Conclusions

The correct identification of both common and rare actionable ALK fusions in advanced NSCLC acquires even more exquisite relevance since a plethora of ALK inhibitors are now available, and the correct treatment approach in the first and further lines may influence the natural history of these patients. The positive response to multiple ALKis of a NSCLC patient carrying a new and rare SPECCL1::ALK fusion serves as a reference for further clinical treatment and encourages the use of both AMP-NGS and in silico protein prediction models to discover and study the oncogenic potential of understudied and rare, potentially druggable ALK fusion variants.

## Data Availability

The raw data supporting the conclusions of this article will be made available by the authors on request.
